# The Anti-Metastatic Role of Aspirin in Cancer: A Systematic Review

**DOI:** 10.3390/ijms27031288

**Published:** 2026-01-28

**Authors:** Rimsha Kanwal, Bilal Jawed, Syed Khuram Zakir, Francesco Gaudio, Riccardo Martinotti, Matteo Botteghi, Stefano Martinotti, Elena Toniato

**Affiliations:** 1 Centre of Advanced Studies and Technology, Department of Innovative Technology in Medicine and Dentistry, University of Chieti, 66100 Chieti, Italy; rimsha.kanwal@phd.unich.it (R.K.); bilal.jawed@phd.unich.it (B.J.); syedkhuramzakir.khuram@phd.unich.it (S.K.Z.); e.toniato@unich.it (E.T.); 2 Unit of Clinical Pathology and Microbiology, Miulli Generale Hospital, 70021 Acquaviva delle Fonti, Italy; 3 Unit of Haematology, F. MiulliUniversity Hospital, LUM University, 70021 Acquaviva delle Fonti, Italy; gaudio@lum.it; 4 Residency Program in Clinical Oncology, Faculty of Medicine, Umberto I University Hospital, University of Rome “La Sapienza”, 00185 Rome, Italy; riccardo.martinotti@apss.tn.it; 5 Experimental Pathology Research Group, Department of Clinical and Molecular Sciences, Universita Politecnica delle Marche, 60121 Ancona, Italy; matteo.botteghi@worldconnex.com; 6 Unit of Clinical Pathology, Department of Medicine and Surgeon, F. Miulli University Hospital, LUM University, Casamassima, 70010 Bari, Italy

**Keywords:** cancer metastasis, aspirin, cyclooxygenase-1 (COX-1), platelet activation, thromboxane A_2_ (TXA2), epithelial mesenchymal transition (EMT), immune modulation

## Abstract

Metastasis is the leading cause of cancer-related mortality. Although aspirin has been associated with reduced metastatic risk, existing evidence is fragmented across experimental systems, and a comprehensive mechanistic synthesis remains lacking. In particular, the relative contributions of platelet aggregation, thromboxane A_2_ (TXA_2_) signaling, and epithelial–mesenchymal transition (EMT) to aspirin’s antimetastatic effects have not been systematically integrated across preclinical and clinical studies. This systematic review was conducted in accordance with PRISMA 2020 guidelines, with the protocol registered in PROSPERO (CRD420251231581). PubMed, Scopus, and Web of Science were searched for studies published between January 2015 and December 2025, alongside ClinicalTrials.gov for completed mechanistic clinical trials. Eligible studies included in vitro, in vivo, and clinical investigations evaluating aspirin or its active metabolite in cancer-related settings and reporting mechanistic outcomes related to metastasis. Clinical studies reporting only survival or incidence outcomes without mechanistic analysis were excluded. The included studies demonstrated that aspirin suppresses metastatic dissemination across multiple cancer types through coordinated platelet-dependent and tumor-intrinsic mechanisms. Aspirin consistently inhibited platelet aggregation and COX-1-dependent TXA_2_ production, disrupting platelet–tumor cell interactions, intravascular metastatic niche formation, and platelet-mediated immune suppression. Clinical mechanistic studies confirmed inhibition of thromboxane biosynthesis and reductions in circulating tumor cells. Beyond platelet effects, aspirin suppressed EMT, migration, and invasion through modulation of EMT transcriptional regulators and inflammatory signaling pathways. Additional mechanisms included activation of AMPK, inhibition of c-MYC signaling, regulation of redox-responsive pathways and impairment of anoikis resistance. This review provides the first integrated mechanistic synthesis of aspirin’s antimetastatic actions across preclinical and clinical evidence, addressing a critical gap in understanding how platelet biology, TXA_2_ signaling, EMT, and tumor-intrinsic survival pathways converge in metastatic suppression. By focusing exclusively on mechanistically informative studies, this work clarifies the biological basis of aspirin’s antimetastatic effects and highlights unresolved questions regarding pathway hierarchy, cancer-type specificity, and translational biomarkers, thereby informing future mechanistic and clinical investigations.

## 1. Introduction

Cancer metastasis, despite substantial advances in cancer diagnosis and treatment, remains the principal cause of cancer-related mortality, accounting for nearly 90% of deaths among patients [1,2]. In 2022, there were an estimated 20 million new cancer cases and 9.7 million cancer-related deaths. The estimated number of individuals alive five years after a cancer diagnosis was 53.5 million. Approximately one in five people will develop cancer in their lifetime, with about one in nine men and one in twelve women dying from the disease [3,4]. In the United Kingdom alone, cancer accounts for more than 150,000 deaths annually [5]. Although targeted therapies directed against oncogenic drivers have improved outcomes in selected patient populations, durable suppression of metastatic progression remains an unmet clinical challenge. Increasing attention has therefore focused on host-dependent mechanisms that facilitate metastatic dissemination and may represent tractable therapeutic targets.

**Figure 1:**
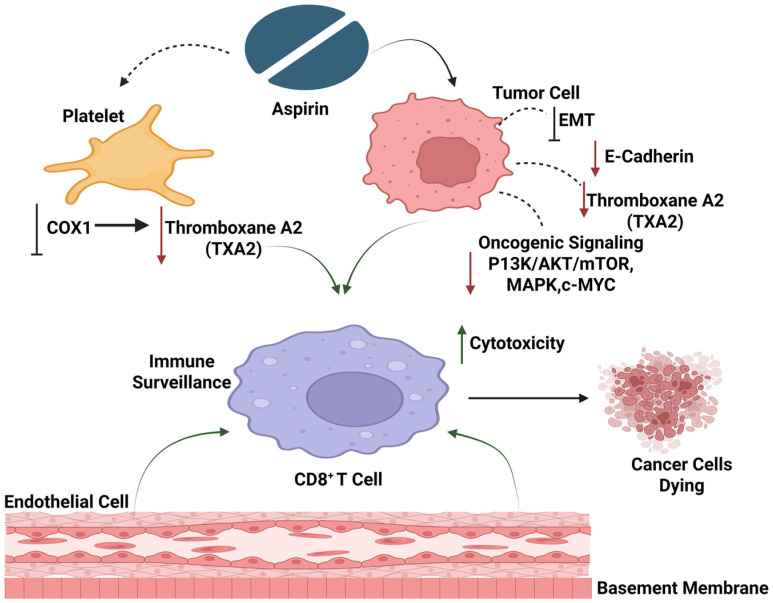
Mechanistic pathway of aspirin-mediated inhibition of cancer progression. Created in BioRender. MARTINOTTI, S. (2026) https://BioRender.com/a8zkwo4 (accessed on 18 November 2025).

Platelets have emerged as critical regulators of tumor progression and metastasis (Figure 1). Beyond their canonical role in hemostasis, platelets actively contribute to early tumorigenesis by shaping an inflammatory tumor microenvironment and by directly interacting with cancer cells [6,7]. Platelet–tumor cell crosstalk induces phenotypic plasticity in cancer cells, including the activation of epithelial–mesenchymal transition (EMT) programs and cyclooxygenase (COX)-1 dependent signaling pathways [8,9]. These interactions promote cancer cell migration, invasion, immune evasion, and survival in circulation, thereby facilitating metastatic spread [10,11]. In turn, cancer cells amplify platelet activation through multiple mechanisms, including enhanced thromboxane A_2_ (TXA_2_) synthesis, establishing a self-reinforcing pro-metastatic loop [12].

TXA_2_ is a bioactive prostanoid generated predominantly by platelet COX-1 and thromboxane synthase and signals through the thromboxane A_2_ receptor (TXA_2_R), a G-protein-coupled receptor expressed primarily in platelets but also in a variety of non-hematopoietic cells [13]. TXA_2_R activation triggers diverse signaling cascades that regulate platelet aggregation, vasoconstriction, inflammation, cell adhesion, motility, proliferation, and survival [14,15,16] Dysregulation of the TXA_2_–TXA_2_R axis has been implicated in multiple malignancies. Elevated expression of TXA_2_R and thromboxane synthase has been associated with enhanced proliferation, migration, invasion, resistance to apoptosis, and metastatic potential in lung, bladder, prostate, and breast cancers [17,18,19]. Increased thromboxane production in human tumors correlates with larger tumor size, hormone receptor negativity, and increased metastatic burden, underscoring its relevance in cancer progression [20].

The importance of prostanoid signaling in metastasis is further supported by studies demonstrating that pharmacological inhibition of COX enzymes or downstream prostanoids markedly suppresses metastatic dissemination in both experimental models and clinical settings [21]. However, the relative contribution of COX-1 versus COX-2, as well as the specific cellular compartments and prostanoids responsible for driving metastasis, remains incompletely resolved [22]. While COX-2–derived prostaglandins such as PGE_2_ have been implicated in tumor-promoting inflammation, platelet-derived COX-1-dependent TXA_2_ appears to play a dominant role during the early stages of hematogenous metastasis, particularly by supporting platelet–tumor cell aggregation, immune evasion, and intravascular niche formation [23].

Aspirin is uniquely positioned to interrogate these mechanisms due to its irreversible inhibition of COX enzymes and its pharmacokinetic properties [24]. At low doses, aspirin preferentially inhibits platelet COX-1, resulting in sustained suppression of TXA_2_ production without substantially affecting COX-2–derived prostanoids in other tissues [25]. Epidemiological studies and post hoc analyses of randomized cardiovascular prevention trials have consistently shown that long-term, low-dose aspirin reduces cancer incidence and mortality, with a particularly pronounced effect on metastatic spread [26]. Notably, reductions in cancer-related deaths have been observed at doses as low as 75–100 mg/day, implicating platelet inhibition rather than systemic anti-inflammatory effects as a key mediator of aspirin’s anticancer activity [27]. Nevertheless, aspirin has also been shown to exert tumor-intrinsic effects, including modulation of EMT, metabolic reprogramming, oxidative stress responses, and anoikis resistance, indicating that its antimetastatic actions may extend beyond platelet inhibition alone [28,29,30].

Despite accumulating evidence linking aspirin, platelet biology, and metastatic suppression, existing studies remain fragmented across experimental systems and cancer types, and no systematic synthesis has comprehensively integrated platelet-dependent and tumor-intrinsic mechanisms. The relative hierarchy of COX-1/TXA_2_ signaling, EMT regulation, immune modulation, and metabolic pathways in mediating aspirin’s antimetastatic effects remains unclear.

In this systematic review, we synthesize preclinical and mechanistic clinical evidence to delineate the biological pathways through which aspirin suppresses cancer metastasis, with a particular focus on platelet aggregation, COX-1/TXA_2_ signaling, and EMT because these pathways represent interconnected, early-stage drivers of metastasis that are directly modulated by aspirin at clinically relevant doses, and for which convergent mechanistic evidence exists across preclinical and clinical studies. By integrating data across experimental models and mechanistically informative clinical studies, this review aims to clarify the mechanistic basis of antimetastatic activity of aspirin. While numerous laboratory studies have elucidated platelet-dependent and tumor-intrinsic mechanisms underlying aspirin’s antimetastatic effects, these pathways have not been systematically examined in clinical settings. This review highlights such translational gaps and emphasizes the need for clinically oriented studies that directly assess these mechanisms in patients.

## 2. Methods

### 2.1. Literature Search Strategy

**Figure 2:**
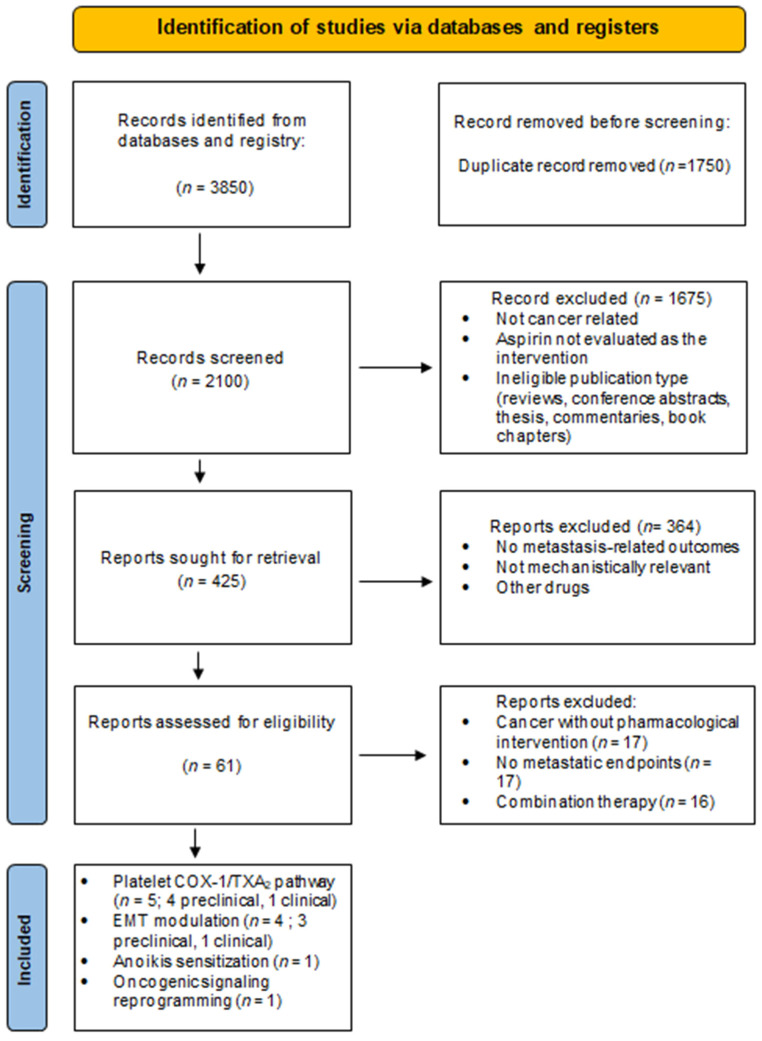
Prisma Flow Diagram.

This systematic review was conducted in accordance with the Preferred Reporting Items for Systematic Reviews and Meta-Analyses (PRISMA 2020) guidelines (Figure 2) [31]. The review protocol was registered in PROSPERO (CRD420251231581) during manuscript preparation. A comprehensive literature search was performed across multiple electronic databases, including PubMed, Scopus, and Web of Science, to identify relevant studies published between January 2015 and December 2025. The search strategy was designed to capture studies evaluating the antimetastatic effects of aspirin and its underlying mechanisms in cancer. Search terms were constructed using a combination of free-text keywords and controlled vocabulary related to aspirin exposure (e.g., “aspirin,” “acetylsalicylic acid,” “salicylate,” “cyclooxygenase inhibitors”), cancer progression (e.g., “cancer,” “tumor,” “metastasis,” “invasion,” “migration”), and metastasis-related mechanisms (e.g., “platelet aggregation,” “thromboxane A_2_,” “epithelial–mesenchymal transition,”). Boolean operators (“AND” “OR”) were systematically applied to combine search concepts and maximize retrieval sensitivity while maintaining specificity. To enhance search completeness and identify relevant clinical evidence, ClinicalTrials.gov was searched for completed clinical trials only, using predefined terms related to aspirin and metastasis-associated outcomes, including metastatic progression, TXA2, EMT and platelet aggregation. The primary objective of the search was to identify studies investigating the antimetastatic effects of aspirin and the mechanistic roles of platelet aggregation, TXA2 and EMT in cancer metastasis.

A total of 3850 records were identified through databases and registry searches. After removal of 1750 duplicate records, 2100 articles remained for title and abstract screening. Of these, 1675 records were excluded for being non-cancer-related, not evaluating aspirin as the intervention of interest, or representing ineligible publication types (reviews, conference abstracts, theses, commentaries, or book chapters). The remaining 425 articles underwent full-text eligibility assessment. Following full-text review, 364 studies were excluded due to the absence of metastasis-related endpoints, lack of mechanistic relevance to platelet aggregation, cyclooxygenase-1/thromboxane A_2_ signaling or epithelial–mesenchymal transition, indirect or unclear aspirin exposure, or inappropriate study design. A total of 61 studies were subjected to detailed qualitative evaluation. Of these, 50 studies were further excluded because they assessed aspirin in non-cancer contexts, evaluated cancer models without aspirin intervention, or lacked sufficient mechanistic or metastatic relevance. Ultimately, 11 studies met all predefined inclusion criteria and were included in the final qualitative synthesis. These comprised 9 preclinical mechanistic studies and 2 mechanistically informative clinical investigations, collectively elucidating the molecular and cellular basis of aspirin-mediated suppression of metastatic competence.

### 2.2. Selection Criteria

Studies were eligible for inclusion if they were original research articles published in English that investigated aspirin (acetylsalicylic acid) or its active metabolite in cancer-related experimental settings. Eligible studies included in vitro and in vivo preclinical models as well as clinical investigations involving human participants. The primary outcome of this review was the antimetastatic effect of aspirin, defined as evidence of reduced metastatic dissemination. Secondary outcomes comprised mechanistic pathways underlying aspirin-mediated suppression of metastasis, including platelet aggregation, cyclooxygenase-1/thromboxane A_2_ signaling, and epithelial–mesenchymal transition. These mechanistic outcomes were considered collectively across the body of evidence and were not required to be assessed within a single study. For clinical studies, inclusion was restricted to investigations that provided mechanistic insight into aspirin’s antimetastatic activity, such as evaluation of platelet-related pathways, thromboxane signaling, epithelial–mesenchymal transition, or related metastasis-associated biological processes. Clinical studies reporting only survival outcomes, metastatic incidence, or disease progression without accompanying mechanistic analysis were not included. This approach ensured consistency with the mechanistic focus of the review. Studies were excluded if they did not evaluate aspirin, focused on aspirin analogs or unrelated interventions, or were not related to cancer. Studies assessing only primary tumor growth, proliferation, apoptosis, or chemo sensitization without relevance to metastatic progression were also excluded. Review articles, editorials, letters, conference abstracts, and case reports were excluded, as were studies not available as full-text articles or published in languages other than English. The restriction to English-language full-text publications was applied to ensure accurate extraction and interpretation of complex mechanistic data.

### 2.3. Screening and Data Extraction

Titles and abstracts of all retrieved records were independently screened against predefined inclusion and exclusion criteria to assess eligibility. Studies considered potentially relevant proceeded to full-text review, where eligibility was reassessed in detail based on study design, aspirin exposure, cancer context, and relevance to metastasis-related or mechanistic outcomes. In addition to database searching, the reference lists of relevant review articles were manually screened to identify additional original research studies evaluating aspirin in metastasis-related mechanistic contexts. Only studies meeting full-text eligibility criteria were considered for inclusion through this process. Screening was performed independently by one reviewer and validated by a second reviewer to ensure reliability. Any discrepancies at either the screening or full-text review stage were resolved through consensus discussion. Data extraction was conducted using a standardized, predefined data extraction form implemented in Microsoft Excel, developed prior to data collection. The extraction form captured key study characteristics, including study design and experimental model (in vitro, in vivo, or clinical), cancer type, aspirin dose and exposure, metastasis-related outcomes, and relevant mechanistic endpoints (e.g., platelet signaling, epithelial–mesenchymal transition, immune modulation). All extracted data were independently verified by a second reviewer to minimize errors and reduce bias. Due to heterogeneity in experimental models, outcome measures, and mechanistic endpoints across included studies, a qualitative synthesis approach was employed. Findings were descriptively summarized, with emphasis on consistent mechanistic patterns, context-dependent effects, and translational relevance across the preclinical and clinical evidence base.

## 3. Results and Discussion

This review summarizes mechanistic evidence from 11 studies published between 2015 and 2025 demonstrating that aspirin suppresses metastatic competence through distinct but interconnected biological pathways (Table 1). Five studies consistently identify platelet COX-1-dependent thromboxane A_2_ (TXA_2_) inhibition as a central mechanism by which aspirin disrupts platelet-mediated tumor cell protection, immune evasion, early intravascular seeding, and metastatic colonization across multiple cancer types. Additional studies show that aspirin attenuates epithelial–mesenchymal transition (EMT)–associated phenotypes, reducing cancer cell migration, invasion, platelet-driven invasiveness, and mesenchymal circulating tumor cell populations. Beyond these effects, aspirin was shown to sensitize tumor cells to anoikis, selectively impairing survival of circulating tumor cells, and to reprogram tumor-intrinsic oncogenic signaling, including activation of the AMPK–NRF2–miR-34 axis with suppression of c-MYC-dependent transcription. Collectively, these findings establish aspirin as a host- and tumor-directed modulator of metastatic fitness, acting primarily through inhibition of platelet signaling, cellular plasticity, and survival pathways.

### 3.1. Aspirin Inhibits Platelet COX-1/TXA_2_-Driven Metastasis and Immune Evasion

A growing body of evidence identifies a reciprocal feed-forward interaction between tumor cells and platelets as a key driver of hematogenous metastasis. Tumor cells activate platelets, while activated platelets promote tumor cell survival in circulation, immune evasion, vascular arrest, and extravasation, thereby facilitating metastatic seeding [43]. In addition to inducing epithelial–mesenchymal transition (EMT), platelets shield circulating tumor cells from immune clearance and enhance their adhesion to the vascular endothelium [44].

Experimental studies provide direct mechanistic support for aspirin-mediated disruption of this process. Guillem-Llobat et al. [32] showed that coculture of HT29 human colon carcinoma cells with platelets induced a mesenchymal-like phenotype characterized by E-cadherin downregulation, *TWIST1* upregulation, increased migratory capacity, and enhanced platelet aggregation. These platelet-induced pro-metastatic changes were abolished by antiplatelet agents, including aspirin, implicating platelet COX-1 signaling in metastatic priming. In vivo, intravenous injection of platelet-primed HT29 cells into NOD-*scid IL2Rγ^null^* mice significantly increased lung metastases, which correlated with elevated systemic thromboxane A_2_ (TXA_2_) and prostaglandin E_2_ (PGE_2_) biosynthesis. Importantly, oral aspirin (20 mg/kg/day; human-equivalent ≈ 150 mg/day), administered before tumor cell injection, prevented platelet-induced metastatic enhancement and suppressed TXA_2_ and PGE_2_ production.

These findings are reinforced by the work of Lucotti et al. [33], who demonstrated that aspirin suppresses lung metastasis across multiple tumor types, including melanoma, breast cancer, and colorectal cancer. Using syngeneic and xenograft models (B16F10, MC-38, 4T1, and MDA-MB-231), medium and high doses of aspirin reduced pulmonary metastatic burden by more than 50%, whereas low-dose aspirin was ineffective. Antimetastatic efficacy correlated with >95% inhibition of platelet COX-1 activity, reflected by reduced serum thromboxane B_2_ (TXB_2_) and impaired COX-1-dependent platelet aggregation, while ADP-mediated aggregation remained unaffected. Genetic deletion or selective pharmacological inhibition of COX-1 phenocopied aspirin’s effects, whereas COX-2 inhibition had no impact on metastatic seeding. Temporal intervention experiments further revealed that aspirin was effective exclusively during the intravascular phase of metastasis, highlighting platelet COX-1/TXA_2_ signaling as a critical determinant of early metastatic survival and lung colonization.

**Figure 3:**
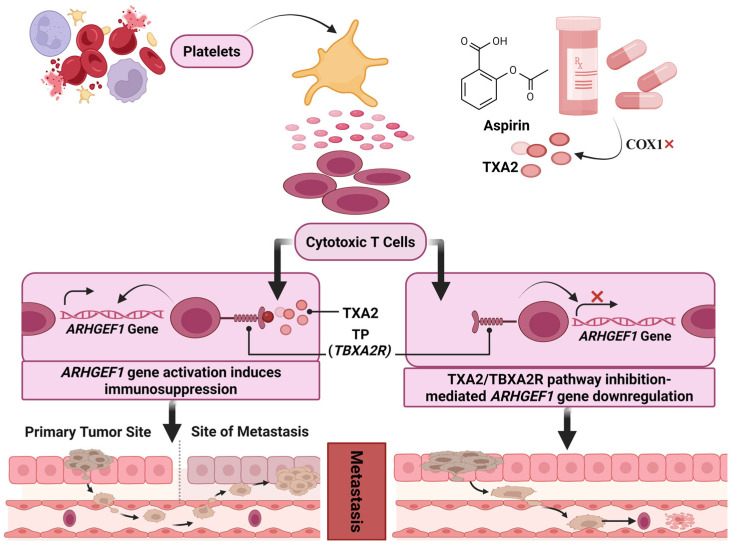
Platelet-derived TXA2 suppresses antitumor immunity by activating ARHGEF1 in T cells, impairing their tumor infiltration. Aspirin inhibits COX-1, reducing TXA2 synthesis and restoring T cell function, which may enhance responses to cancer therapy. Created in BioRender. MARTINOTTI, S. (2026) https://BioRender.com/9dvbtnt (accessed on 18 November 2025).

Beyond its effects on platelet–tumor interactions, TXA_2_ also functions as a potent immunosuppressive mediator during early metastatic dissemination. Yang et al. [34] demonstrated that platelet-derived TXA_2_ suppresses antitumor T-cell immunity via activation of an ARHGEF1-dependent inhibitory pathway, impairing T-cell receptor signaling, proliferation, and effector function. Conditional deletion of Arhgef1 in T cells enhanced immune-mediated rejection of both lung and liver metastases, while aspirin or selective COX-1 inhibition significantly reduced metastatic burden in a T-cell-dependent manner (Figure 3). These findings establish platelet TXA_2_ as a previously unrecognized immune checkpoint operative during early metastatic seeding. In addition to platelet-derived TXA_2_–mediated immune suppression, aspirin has been shown to counteract tumor immune evasion through TXA_2_-independent mechanisms. Tumor-derived exosomes and PD-L1 signaling are established mediators of systemic immune suppression that facilitate tumor persistence and progression. Wang et al. [45] demonstrated that low-dose aspirin suppresses radiotherapy-induced release of immunosuppressive exosomes in breast cancer, restoring NK-cell proliferation and enhancing antitumor immunity in vivo. Similarly, Xiao et al. [46] showed that aspirin epigenetically downregulates PD-L1 expression by inhibiting KAT5-dependent histone acetylation, thereby restoring T-cell activation and improving responsiveness to immune checkpoint blockade. Although these studies do not directly assess metastatic dissemination, they reinforce a unifying concept emerging from platelet COX-1/TXA_2_ models: aspirin alleviates tumor-driven immune suppression at early stages of disease progression, creating conditions that are unfavorable for metastatic survival and outgrowth.

Integrating transcriptional and immune mechanisms, Zou et al. [35] identified upregulation of Tbxas1 (thromboxane synthase-1) as a conserved feature of highly metastatic thyroid cancer models. Highly metastatic derivatives exhibited activation of inflammatory, immunosuppressive, EMT-associated, and stemness-related pathways. Low-dose aspirin (25 mg/kg, three times weekly) significantly reduced both the number and size of lung metastatic foci (6.8 ± 1.5 vs. 25 ± 3.4, *p* < 0.01) following intravenous tumor cell injection. This reduction was accompanied by suppression of MAPK and AKT signaling, reduced PD-L1 expression, partial reversal of EMT, and attenuation of cancer stem–like features, confirming a functional role for TXA_2_-dependent platelet signaling in metastatic immune escape and colonization. The clinical relevance of the aspirin mediated suppression of TXA_2_ was explored in a mechanistic sub-study of the Add-Aspirin trial [36]. Using urinary 11-dehydro-thromboxane B_2_ (U-TXM) as a biomarker of in vivo platelet activation, the investigators identified persistently elevated TXA_2_ biosynthesis in 716 patients with non-metastatic breast, colorectal, gastro-esophageal, or prostate cancer following radical therapy. Baseline U-TXM concentrations substantially exceeded those of healthy controls (~500 pg/mg creatinine), with the highest levels observed in gastro-esophageal (1675 pg/mg) and colorectal (1060 pg/mg) cancers. Elevated U-TXM correlated with systemic inflammatory markers, suggesting the presence of a chronic platelet-driven inflammatory and immunosuppressive milieu in cancer survivors. Importantly, Hogan et al. [36] demonstrated that low-dose aspirin (100 mg/day) consistently reduced U-TXM by 77–82% across all tumor types, whereas escalation to 300 mg/day conferred no additional suppression of systemic TXA_2_ production. These findings confirm that standard low-dose aspirin is sufficient to inhibit the systemic TXA_2_ axis implicated in T-cell suppression, thereby functionally aligning clinical biomarker responses with the mechanistic pathway delineated by Yang et al. [34]. These findings are consistent with previous evidence in colorectal cancer showing that the survival benefit of aspirin appears to be limited to tumors with high HLA class I expression, suggesting that immune-related biomarkers may help identify patients who derive the greatest advantage from aspirin therapy [47]. Importantly, platelet count has also been adopted as a prognostic biomarker and a treatment-selection criterion in patients at risk of hepatocellular carcinoma and in those with viral cirrhosis, reinforcing the broader clinical relevance of platelet-driven biology in cancer progression [48,49]. This highlights the need for robust biomarker-led stratification within prospective randomized controlled trials to define the cancer types and patient populations in which aspirin is most effective, particularly in view of the inconsistent results reported across different patient subgroups [50,51].

Dose-dependent pharmacodynamics within tumor tissue were further delineated by Patrignani et al. [52]. Although all aspirin regimens achieved near-complete platelet COX-1 acetylation, only higher doses (300 mg once daily or 100 mg twice daily) significantly acetylated tumor COX-1 and reduced intratumoral TXB_2_ production. In contrast, low-dose aspirin was associated with upregulation of pro-metastatic markers such as *VIM* and *TWIST1*, underscoring distinct effects on platelet versus tumor compartments and suggesting that platelet-mediated antimetastatic benefits may be achieved at lower doses than those required to modulate tumor-intrinsic pathways.

Population-based analyses further support the role of TXA_2_ in lethal disease. Elevated urinary TXB_2_ was inversely associated with aspirin use and strongly associated with metastatic prostate cancer and cancer-specific mortality in African American men, but not European American men. High TXB_2_ levels correlated with increased prostate cancer risk (OR = 1.50), metastatic disease (OR = 2.60), and prostate cancer-specific mortality (HR = 4.74), identifying population-specific vulnerability to TXA_2_-driven metastatic progression and supporting TXA_2_ inhibition as a chemo-preventive strategy in high-risk groups [53].

Collectively, these data establish platelet-derived TXA_2_ signaling as a central regulator of metastatic dissemination, immune evasion, and niche formation. They provide a unified mechanistic framework explaining aspirin’s antimetastatic activity across multiple cancer types and underscore the importance of biomarker-guided patient stratification in future clinical trials. Targeting the platelet COX-1/TXA_2_ axis represents a rational and clinically actionable strategy to limit metastatic progression, particularly in patient populations characterized by heightened platelet activation and immune suppression.

### 3.2. Aspirin Reprograms Oncogenic Signaling Networks to Suppress Metastatic Competence

**Figure 4:**
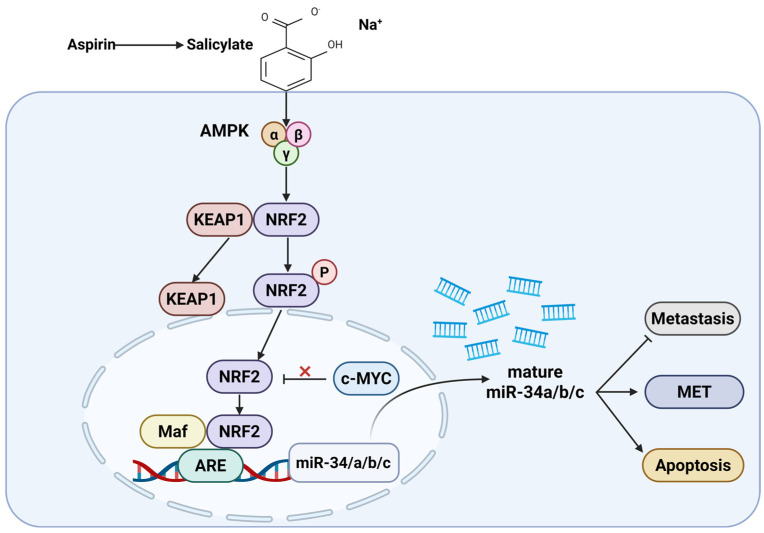
Salicylate, the active aspirin metabolite, activates AMPK, leading to NRF2 release from KEAP1 and its nuclear translocation. Nuclear NRF2 partners with Maf proteins to activate ARE-driven transcription of the miR-34a/b/c cluster, while AMPK-dependent suppression of c-MYC further enhances this pathway, resulting in increased MET, apoptosis, and inhibition of metastatic progression. Created in BioRender. MARTINOTTI, S. (2026) https://BioRender.com/a8zkwo4 (accessed on 18 November 2025).

Liu et al. [37] demonstrated that aspirin alters oncogenic signaling programs directly relevant to metastatic progression. Salicylate, the active metabolite of aspirin, induces the tumor-suppressive microRNAs miR-34a and miR-34b/c in a p53-independent manner through activation of AMPK, which in turn activates NRF2 to drive miR-34 transcription, bypassing the canonical KEAP1 inhibitory axis. In parallel, salicylate suppresses c-MYC an established inhibitor of NRF2-dependent transcription—in an AMPK-dependent manner, and this repression is required for efficient miR-34 induction. Within the nucleus, NRF2 cooperates with Maf proteins to initiate antioxidant response element (ARE)–mediated transcription of the miR-34 family. Functional loss of miR-34a/b/c markedly attenuated salicylate’s ability to suppress colorectal cancer migration, invasion, and metastatic dissemination, establishing the AMPK–NRF2–miR-34 axis as a central antimetastatic mechanism (Figure 4). This study employed a broad panel of colorectal cancer cell lines, including HCT116 (p53 wild-type and p53-null), RKO (p53 wild-type and p53-null), SW48, SW480, DLD-1, SW620, and luciferase-expressing SW620-Luc2 cells, alongside non-transformed human colonic epithelial cells (HCEC-1CT) and colon fibroblasts (CCD-18Co). Treatment with salicylate at 5 mM significantly suppressed CRC cell proliferation and induced apoptosis in a p53-independent manner while sparing non-transformed intestinal cells. Salicylate also inhibited migration and invasion and promoted a mesenchymal-to-epithelial transition (MET), characterized by downregulation of Vimentin and SNAIL and upregulation of E-cadherin. Mechanistically, AMPK–NRF2 activation and concomitant c-MYC repression were required for apoptosis induction, MET, and suppression of migratory behavior, as genetic ablation or inhibition of miR-34a/b/c significantly attenuated these effects. In vivo, pretreatment of SW620-Luc2 cells with salicylate completely abolished lung metastasis formation following tail-vein injection into NOD/SCID mice, with metastatic suppression partially reversed by miR-34a antagonism, confirming a miR-34-dependent antimetastatic mechanism downstream of aspirin metabolism.

Consistent with this mechanism, previous studies have shown that salicylate suppresses c-MYC expression [54], and genetic evidence further strengthens this link. The single-nucleotide polymorphism rs6983267 at chromosome 8q24, which modulates c-MYC expression, has been associated with variability in aspirin’s chemo-preventive benefit [55]. Individuals carrying the T allele—associated with reduced c-MYC expression due to weaker binding of the WNT-responsive transcription factor TCF4—derive a more pronounced protective effect from aspirin [56,57]. Given the high prevalence of this allele in European populations (~49%), a substantial proportion of individuals may be genetically predisposed to benefit from aspirin’s ability to suppress c-MYC driven oncogenic signaling [58]. In addition to miR-34-dependent tumor reprogramming, aspirin also modulates oncogenic signaling pathways that underpin metastatic competence and therapeutic resistance. In pancreatic cancer, where constitutive PI3K/AKT/mTOR activation drives epithelial–mesenchymal transition and chemoresistance, aspirin enhanced the antitumor efficacy of gemcitabine by suppressing proliferation, migration, and invasion while promoting apoptosis. These effects were associated with inhibition of PI3K/AKT/mTOR signaling and partial reversal of EMT, reinforcing the concept that aspirin constrains metastatic progression through coordinated reprogramming of oncogenic networks sustaining tumor aggressiveness [59].

### 3.3. Aspirin Targets TXA_2_-Mediated Anoikis Resistance in Metastatic Cells

**Figure 5:**
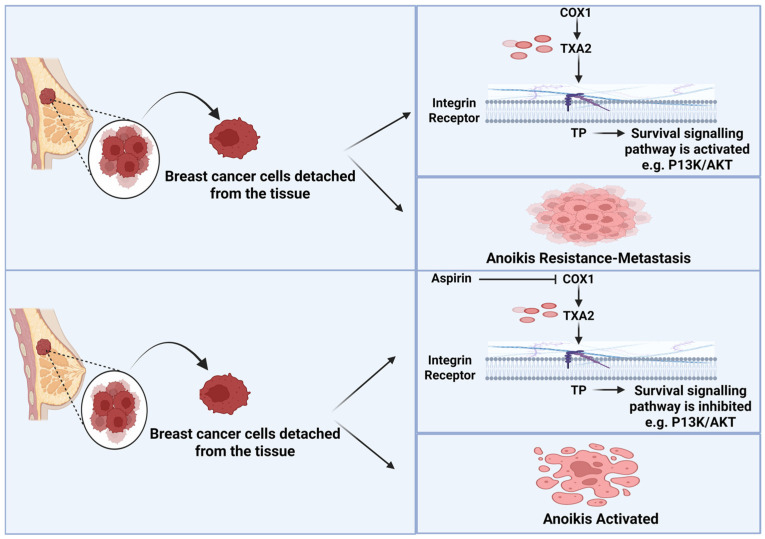
Aspirin suppresses COX-1-dependent thromboxane A_2_ (TXA_2_) production, reducing activation of the thromboxane receptor (TP) in detached cancer cells. Diminished TP-AKT signaling restores anoikis sensitivity and limits metastatic survival. Created in BioRender. MARTINOTTI, S. (2026) https://BioRender.com/9dvbtnt (accessed on 18 November 2025).

Anoikis resistance develops when cells that lose matrix attachment circumvent apoptotic signaling through a series of molecular and biochemical adaptations. These alterations confer the capacity for survival in circulation and are strongly associated with increased invasiveness, metastatic dissemination, therapeutic resistance, and tumor recurrence [60,61,62]. Xu et al. [38] study found that aspirin inhibits anoikis resistance in breast cancer, thereby suppressing metastatic dissemination (Figure 5). This study examined thromboxane A_2_ (TXA_2_)-driven breast cancer metastasis using human (MDA-MB-231) and murine (4T1 and E0771) breast cancer cell lines in syngeneic BALB/c and C57BL/6 mouse models, including thromboxane A_2_ receptor–deficient (TP^−^/^−^) mice. Aspirin was administered orally at 100 or 200 mg/kg daily in vivo and was well tolerated. Aspirin treatment significantly reduced lung metastatic nodules and circulating tumor cells, with only modest effects on primary tumor growth, indicating a preferential inhibition of hematogenous dissemination. Mechanistically, circulating and detached tumor cells showed increased expression of COX-1, COX-2, TBXAS1, and TP, linking activation of the TXA_2_ pathway to metastatic competence. Pharmacological COX inhibition, genetic silencing of TP in tumor cells, or host TP deficiency sensitized breast cancer cells to anoikis and markedly attenuated lung metastasis. TXA_2_ signaling promoted metastatic survival by sustaining AKT activation under anchorage-independent conditions, whereas aspirin suppressed TXA_2_ production and disrupted TP–AKT signaling, leading to apoptotic cell death. These findings identify the TXA_2_–TP–AKT axis as a key mediator of breast cancer lung metastasis and provide mechanistic evidence supporting aspirin’s antimetastatic effects via inhibition of TXA_2_ pathway as a central regulator of anoikis resistance.

### 3.4. Aspirin Suppresses EMT-Driven Metastatic Programs

The initiation of tumor metastasis is characterized by enhanced migratory and invasive capacities, which are largely enabled by epithelial–mesenchymal transition (EMT). During tumor progression, EMT is marked by loss of epithelial characteristics, such as E-cadherin expression, and acquisition of mesenchymal markers, including vimentin, thereby facilitating dissemination and colonization of distant organs [63,64,65].

Preclinical evidence demonstrates that aspirin suppresses inflammation-driven EMT and metastatic progression. Ying et al. [39] showed that lipopolysaccharide (LPS; 10 µg/mL) markedly enhanced migration, invasion, and EMT in murine C26 and human HCT116 colorectal cancer cells, accompanied by E-cadherin downregulation and vimentin upregulation. In vivo, splenic vein injection of C26 cells into syngeneic BALB/c mice resulted in extensive liver metastasis, which was significantly reduced by aspirin treatment. Aspirin pretreatment (10 mM in vitro; systemically administered in vivo) effectively abrogated LPS-induced metastatic capacity. Mechanistically, LPS-driven EMT and metastasis were mediated through Toll-like receptor 4 (TLR4)-dependent activation of NF-κB signaling, while aspirin downregulated TLR4 expression, inhibited NF-κB activation, and reversed EMT phenotypes. Genetic silencing of TLR4 or pharmacological inhibition of NF-κB phenocopied aspirin’s antimetastatic effects, establishing the TLR4–NF-κB–EMT axis as a critical target of aspirin in inflammation-associated metastasis.

Complementary mechanistic insights were provided by Khan et al. [40] in oncogenic KRAS-driven non-small cell lung cancer (NSCLC). Using human NSCLC cell lines A549 (KRAS G12 mutant), H1299 (KRAS wild-type), and NCI-H522 (KRAS mutant), aspirin at a clinically relevant concentration (2.5 mM) significantly reduced cancer cell migration. Aspirin restored E-cadherin expression and suppressed EMT regulators, including Slug, vimentin, Twist, MMP-2, and MMP-9. These effects were mediated through inhibition of NF-κB signaling, evidenced by reduced IκBα phosphorylation, impaired p65 nuclear translocation, and diminished NF-κB occupancy at the SLUG promoter. By disrupting the KRAS–MEK/ERK–Elk-1–NF-κB–Slug axis, aspirin transcriptionally repressed Slug and reversed EMT. Although this study did not include in vivo metastasis models, it provides strong mechanistic evidence that aspirin constrains EMT-driven metastatic competence in KRAS-mutant lung cancer cells.

Circulating tumor cells (CTCs) rely on sustained EMT programs to survive in circulation and seed distant metastases [66,67]. This process is driven by soluble factors derived from stromal and blood components, including growth factors, cytokines, and proteases [68,69]. Activated platelets are particularly important contributors, providing pro-metastatic signals that induce EMT and enhance tumor cell invasiveness [70]. Cooke et al. [41] investigated platelet-driven pro-metastatic behavior in ovarian cancer using two ascites-derived metastatic cell lines, SK-OV-3 and 59 M. Platelets preferentially adhered to and activated in response to SK-OV-3 cells, inducing EMT-like morphological changes and transcriptional reprogramming characterized by E-cadherin downregulation and increased expression of mesenchymal markers (vimentin, PAI-1, *SNAI1*, *JAG1*). Functionally, platelet co-culture increased SK-OV-3 invasion 3.5–3.8-fold, demonstrating a direct pro-invasive role of platelets. Antiplatelet treatment significantly attenuated this effect: COX-1 inhibition with aspirin (20 µM) and P2Y12 inhibition with 2MeSAMP (50 µM) reduced platelet-mediated invasion, with P2Y12 blockade showing greater efficacy. Notably, aspirin did not prevent platelet adhesion or EMT gene induction but selectively impaired platelet-driven invasive capacity, indicating that platelet signaling downstream of adhesion—rather than EMT initiation alone—drives metastatic behavior. Functionally, platelet–tumor cell interactions have been shown to directly promote metastatic dissemination. Supporting this concept, Labelle et al. [71] demonstrated using murine tumor cell lines and in vivo mouse models that platelet-derived transforming growth factor-β (TGF-β), together with direct platelet–tumor cell contact, induces EMT programs and enhances tumor cell extravasation and metastatic seeding. These findings provide a mechanistic framework for the antimetastatic effects of antiplatelet strategies, including aspirin. Collectively, these findings establish platelet–tumor cell interactions as key drivers of EMT, extravasation, and metastatic seeding. Consistent with this mechanistic framework, thrombocytosis is clinically associated with adverse cancer outcomes, including increased metastatic risk and reduced survival. Conversely, antiplatelet interventions—most notably aspirin—have been linked to a reduced incidence of metastasis and improved prognosis across multiple malignancies [72,73,74].

Clinical relevance for EMT modulation by aspirin is supported by a phase II trial (NCT02602938) conducted by Yang et al. [42]. In this study, 21 patients with metastatic colorectal cancer (MCC) and 19 patients with metastatic breast cancer (MBC) received low-dose aspirin (100 mg/day) for 2 months. Aspirin treatment led to a significant reduction in total CTC counts in MCC patients, whereas no significant change was observed in MBC patients. Importantly, aspirin selectively reduced the mesenchymal (M^+^) CTC subpopulation and increased the relative proportion of epithelial CTCs in MCC. Consistent with EMT suppression, vimentin expression was significantly decreased in mesenchymal and bio phenotypic CTCs, particularly in patients who exhibited an overall decline in CTC numbers. These findings provide clinical evidence that aspirin modulates EMT-associated phenotypes in circulating tumor cells, supporting its role in limiting metastatic competence rather than directly inducing tumor regression.

Collectively, these experimental and clinical studies demonstrate that aspirin suppresses EMT-driven metastatic programs across multiple cancer contexts by targeting inflammatory signaling, oncogenic transcriptional networks, and platelet-mediated pro-invasive cues. This convergence of evidence underscores aspirin’s potential as a clinically actionable antimetastatic agent through modulation of EMT and metastatic competence.

## 4. Limitations and Future Directions

This systematic review integrates mechanistic evidence from preclinical and mechanistically informative clinical studies examining aspirin’s antimetastatic effects, with a focus on platelet-derived TXA_2_ signaling, immune modulation, epithelial–mesenchymal transition, anoikis sensitization, and miRNA-mediated reprogramming. The evidence base is largely derived from in vitro and animal models, which, although essential for mechanistic insight, cannot fully capture tumor heterogeneity, human pharmacokinetics, or the complexity of the tumor microenvironment, thereby limiting direct clinical extrapolation. Available clinical studies are few and typically small, short-term, and biomarker-driven, with limited assessment of long-term outcomes such as metastasis-free or overall survival. Moreover, heterogeneity across cancer types, experimental models, dosing regimens, and genetic backgrounds complicates generalization and leaves the optimal aspirin dose unresolved, particularly with respect to platelet-dependent versus tumor-intrinsic effects. Given this diversity, quantitative meta-analysis and formal risk-of-bias tools were not appropriate; instead, a qualitative synthesis was undertaken with explicit consideration of model-specific constraints and translational relevance. Although the search strategy was comprehensive and included multiple databases and ClinicalTrials.gov, restriction to English-language publications and the absence of mechanistic endpoints or published results in several registered trials may have limited inclusion. Consequently, while the review provides a robust mechanistic framework supporting aspirin’s antimetastatic potential, definitive conclusions regarding clinical efficacy across cancer types cannot yet be drawn. Future research should therefore prioritize mechanistically driven clinical studies incorporating standardized dosing, biomarker-guided patient stratification, and metastasis-related outcomes to bridge these translational gaps.

## 5. Conclusions

This systematic review synthesizes mechanistic evidence indicating that aspirin exerts anti metastatic effects through coordinated platelet-dependent and tumor-intrinsic pathways, including inhibition of TXA_2_ signaling, modulation of immune responses, suppression of epithelial–mesenchymal transition, sensitization to anoikis, and miRNA-mediated transcriptional reprogramming. Collectively, these findings provide a coherent biological framework supporting aspirin’s role in limiting metastatic competence. However, given the predominance of preclinical evidence and the limited availability of mechanistically informative clinical studies, definitive conclusions regarding clinical efficacy across cancer types cannot yet be drawn. Nevertheless, the consistency of mechanistic signals across experimental systems supports further investigation of aspirin as a low-cost adjunct in oncology and underscores the need for rigorously designed, biomarker-driven clinical studies to translate these insights into patient benefit.

## Figures and Tables

**Table 1:** Mechanistic Evidence Supporting Aspirin’s Antimetastatic Activity Across Cancer Types.

Mechanism/Pathway	Cancer Type	Model/Patient	Key Findings and Outcomes	Dosing	Reference
Platelet COX1/TXA_2_ Inhibition	Colorectal Cancer	Human colon carcinoma cell line HT29, NOD-*scid IL2Rγ^null^* (NSG)	Aspirin abrogated platelet-induced EMT, tumor cell–induced platelet aggregation, and lung metastatic burden by suppressing platelet COX-1–derived TXA_2_/PGE_2_ biosynthesis	Aspirin 20 mg/kg/d orally	Guillem-Llobat et al. [32]
	Melanoma, breast cancer, colorectal cancer	syngeneic C57BL/6 and BALB/c mouse models bearing B16F10 melanoma, MC-38 colon carcinoma, and 4T1 and MDA-MB-231 breast cancer cells, complemented by COX-1–deficient, platelet-depleted, and *Cx3cr1^GFP/+^* reporter mice to delineate platelet COX-1/TXA_2_-dependent mechanisms	Aspirin suppressed lung metastasis across tumor types by inhibiting platelet COX-1/TXA_2_ signaling, reducing platelet aggregation, tumor cell retention, endothelial activation, and monocyte recruitment during early metastatic seeding	Aspirin was administered via drinking water at 30 mg/L (low dose), 180 mg/L (medium dose), and 625 mg/L (high dose),	Lucotti et al. [33]
	Lung, liver, colon, breast	Genetically engineered mouse models with T cell–specific Arhgef1 deletion; syngeneic IV tumor injection models	Aspirin restored CD8^+^ T-cell activation by blocking platelet-derived TXA_2_–ARHGEF1 immunosuppressive signaling, resulting in immune-mediated clearance of lung and liver metastases	Aspirin 600 mg/L in drinking water	Yang et al. [34]
	Thyroid	Athymic BALB/c-nu/nu mice	Low-dose aspirin reduced lung metastatic burden by suppressing Tbxas1-dependent TXA_2_ production, platelet aggregation, EMT, MAPK/AKT signaling, stemness features, and immune checkpoint expression	Low-dose aspirin 25 mg/kg, 3× weekly	Zou et al. [35]
	Breast, colorectal, gastro-esophageal, prostate	716 patients post-radical therapy	Low dose aspirin reduced U-TXM level from 77–82%; higher doses no additional benefit	Low-dose aspirin 100 mg/day; 300 mg/day also studied	Hogan et al. [36]
Oncogenic Modulation	Colorectal	HCT116 and SW480 cell lines; xenograft mouse model	Salicylate upregulates miR-34a/b/c via AMPK → suppresses c-MYC; inhibits migration, invasion and metastasis	Salicylate 1–5 mM in vitro; 200 mg/kg/day in vivo	Liu et al. [37]
Anoikis Sensitization	Breast	Breast cancer cell lines MDA-MB-231, E0771and 4T1	Aspirin inhibits AKT/ERK signaling, inhibit anoikis resistant increases apoptosis; reduces lung metastases	100, 200 mg/kg/day	Xu et al. [38]
EMT Pathway Inhibition	Colon Cancer Cells	Murine C26 and human HCT116 colon cancer cell lines, Syngeneic BALB/c mice	Aspirin inhibits LPS-induced EMT and metastatic potential of colon cancer cells by downregulating TLR4/NF-κB signaling, reducing migration, invasion, and liver metastasis.	10 mM	Ying et al. [39]
	Non-small cell lung carcinoma	Human NSCLC cell lines: A549 (KRAS G12 mutant), H1299, NCI-H522	Aspirin suppresses EMT-associated migration by downregulating Slug, restoring E-cadherin, and inhibiting NF-κB signaling, indicating reduced metastatic competence	2.5 mM	Khan et al. [40]
	Ovarian Cancer Cell	Human metastatic ovarian cancer cell lines SK-OV-3 and 59 M co-cultured with human platelets (in vitro)	Platelets induced EMT-like gene expression and increased SK-OV-3 invasion by 3.5–3.8-fold, while aspirin significantly reduced platelet-mediated invasion via COX-1 inhibition without affecting platelet adhesion or EMT gene induction, indicating suppression of EMT-associated invasive function rather than EMT initiation.	20 µM	Cooke et al. [41]
	Metastatic colorectal cancer (MCC); Metastatic breast cancer (MBC)	Phase II clinical trial; 21 MCC patients and 19 MBC patients	Aspirin treatment significantly reduced total circulating tumor cell (CTC) counts in metastatic colorectal cancer and selectively decreased mesenchymal (EMT-associated) CTCs with lower vimentin expression, indicating attenuation of EMT-linked metastatic potential; these effects were not significant in metastatic breast cancer.	Aspirin 100 mg/day orally for 2 months	Yang et al. [42]

## Data Availability

No new data were created or analyzed in this study. Data sharing is not applicable to this article.
